# Recently integrated *Alu* insertions in the squirrel monkey (*Saimiri*) lineage and application for population analyses

**DOI:** 10.1186/s13100-018-0114-7

**Published:** 2018-02-12

**Authors:** Jasmine N. Baker, Jerilyn A. Walker, Michael W. Denham, Charles D. Loupe, Mark A. Batzer

**Affiliations:** Department of Biological Sciences, Louisiana State University, 202 Life Sciences Bldg., Baton Rouge, LA 70803 USA

**Keywords:** Retroposon, *Saimiri*, *Alu* polymorphism, Population structure

## Abstract

**Background:**

The evolution of *Alu* elements has been ongoing in primate lineages and *Alu* insertion polymorphisms are widely used in phylogenetic and population genetics studies. *Alu* subfamilies in the squirrel monkey (*Saimiri*), a New World Monkey (NWM), were recently reported. Squirrel monkeys are commonly used in biomedical research and often require species identification. The purpose of this study was two-fold: 1) Perform locus-specific PCR analyses on recently integrated *Alu* insertions in *Saimiri* to determine their amplification dynamics, and 2) Identify a subset of *Alu* insertion polymorphisms with species informative allele frequency distributions between the *Saimiri sciureus* and *Saimiri boliviensis* groups.

**Results:**

PCR analyses were performed on a DNA panel of 32 squirrel monkey individuals for 382 *Alu* insertion events ≤2% diverged from 46 different *Alu* subfamily consensus sequences, 25 *Saimiri* specific and 21 NWM specific *Alu* subfamilies. Of the 382 loci, 110 were polymorphic for presence / absence among squirrel monkey individuals, 35 elements from 14 different *Saimiri* specific *Alu* subfamilies and 75 elements from 19 different NWM specific *Alu* subfamilies (13 of 46 subfamilies analyzed did not contain polymorphic insertions). Of the 110 *Alu* insertion polymorphisms, 51 had species informative allele frequency distributions between *Saimiri sciureus* and *Saimiri boliviensis* groups.

**Conclusions:**

This study confirms the evolution of *Alu* subfamilies in *Saimiri* and provides evidence for an ongoing and prolific expansion of these elements in *Saimiri* with many active subfamilies concurrently propagating. The subset of polymorphic *Alu* insertions with species informative allele frequency distribution between *Saimiri sciureus* and *Saimiri boliviensis* will be instructive for specimen identification and conservation biology.

**Electronic supplementary material:**

The online version of this article (10.1186/s13100-018-0114-7) contains supplementary material, which is available to authorized users.

## Background

Short interspersed elements (SINEs) have been key mobile elements in genomic studies and have helped researchers delve into the structure and history of the genomes which they reside [1–6]. SINEs, specifically *Alu* elements, have been extremely important in understanding genomic diversity, systematics and phylogenomics within primates [7–13]. They have been shown to shape the structure of primate genomes [14] and play an important role in phylogenetic studies of primates [11, 13–21]. *Alu* elements are non-autonomous, non-long terminal repeat retrotransposons found in primate genomes. They are commonly used for these analyses due to their primate specificity, small size (~ 300 base pairs) and unidirectional mode of evolution [22–26]. Since they are unidirectional insertions, they allow for confident inference that the ancestral state of an element is the absence of that element for each locus under examination [27].

The squirrel monkey (genus *Saimiri*) is a small forest dwelling neotropical primate native to Central and South America that belongs to the family Cebidae. Squirrel monkeys are commonly used in biomedical research [28–30] since they have similar immune systems to humans. In addition, squirrel monkeys are small and more easily handled compared to large Old World primates such as the rhesus macaque and chimpanzee. Some of the biomedical studies focus on infectious disease, gene expression, cancer treatments, reproductive physiology, and viruses [31–37]. Species differences with regard to disease susceptibility has largely been overlooked until recently [38].

Prior to 1984, squirrel monkeys were considered a single species, *Saimiri sciureus*, with many subspecies geographically separated [28]. In 1984, Hershkovitz published a detailed taxonomy of squirrel monkeys. Hershkovitz divided *Saimiri* into two major groups, *Saimiri boliviensis* and *Saimiri sciureus* [39]. The *S. boliviensis* group has one species that is subdivided into two subspecies, *S. boliviensis boliviensis* and *S. peruviensis*. The *S. sciureus* group consists of three species, *S. sciureus*, *S. oerstedii* and *S. ustus*, with the former two species harboring six subspecies [39, 40]. Subsequent [41, 42] studies using molecular and genetic data have generally supported this classification system. The samples used in this study represented both major groups as well as subspecies *S. boliviensis peruviensis, S. oerstedii oerstedii, and S. sciureus macrodon* (Additional file 1). Given recent nomenclature changes, it is not surprising that some tissue samples or specimens from older studies/stocks in natural science museums may be labeled simply as *Saimiri*, squirrel monkey, or *S. sciureus*. This does not mean the samples are necessarily mislabeled, but more likely represent incomplete identification due to limited availability regarding source animal data at the time of sampling. Studies to develop systems for *Saimiri* species identification have attempted to resolve this issue [40–44] by using various types of genetic markers. Therefore, having more nuclear autosomal genetic markers, especially those which are identical by descent, such as *Alu* element retrotransposon insertions would increase the number of species informative genetic markers.

Few studies have been conducted on mobile element dynamics within New World primates; however, the studies available have provided great insight into their genomes. Specifically, *Alu* elements have given a good representation of genome evolution within and between species. *Alu* elements have been used to confirm family relationships between New World monkeys (NWM) [10, 11, 43, 45]. New World monkeys have been shown to have platyrrhine specific *Alu* element subfamilies--*Alu*Ta7, *Alu*Ta10, and *Alu*Ta15 [10]. These subfamilies have amplified throughout the NWM lineage and have shown *Cebus* and *Sapajus* are sister taxa [46]. New world monkey specific subfamilies have also been used to investigate hybridization within the *Saimiri* lineage [47] and for use as identification markers [43].

A detailed *Alu* subfamily analysis of *Saimiri* was recently reported by Baker et al. [48]. In that study on the evolution of *Alu* subfamilies in the *Saimiri* lineage [48], 108 *Alu* subfamilies within the genome [saiBol1], with 46 of those unique to the *Saimiri* lineage and the other 62 being NWM subfamilies [10, 49], were reported. These subfamilies were defined based on diagnostic nucleotide substitutions, insertions, or deletions that were exclusively shared. Nearly half of the *Alu* subfamilies included members that appeared to be relatively young insertion events (≤ 2% sequence divergence from their respective consensus sequence).

The purposes of this study were to identify polymorphic *Alu* insertions to examine population structure in *Saimiri* and to identify recently integrated insertions that might be informative for species identification. To accomplish these goals, we targeted recently integrated insertions and designed locus specific PCR primers for at least five *Alu* elements from every subfamily that was identified as ‘young’.

## Methods

### *Alu* element ascertainment

A data set of full length *Alu* elements from the *Saimiri* genome [saiBol1] was generated by using the Blat Table Browser. *Alu* full length elements plus 600 base pairs (bp) of flanking were obtained from the University of California Santa Cruz (UCSC) table browser. Full length elements are described as beginning within 4 bp of its respective consensus sequence and being ≥267 bp. *Saimiri* specific elements were RepeatMasked using an in-house installation of RepeatMasker [50] to determine the percent sequence divergence compared to their respective consensus sequences. Young elements, defined here as having a sequence divergence of ≤2% were retained for further analyses. We targeted at least five *Alu* elements for experimental validation from each *Alu* subfamily computationally determined to contain young elements.

### Oligonucleotide primer design

Orthologous sequences to each respective *Alu* plus flanking were retrieved from human [hg38] and marmoset [calJac3] genomes using BLAT [51]. A multiple sequence alignment was created for each locus using BioEdit [52]. Oligonucleotide primers for polymerase chain reaction (PCR) were designed using Primer3 [53, 54] with the following adjustments: Tm range = 57–62, Max TmDifference =2, max poly x = 3, min Gc content = 40. All primers were ordered from Sigma Aldrich (Woodlands, TX). A list of PCR primers and genomic locations is available in Additional file 1.

### DNA samples

A list of *Saimiri* samples and their source information is available in Additional file 1 (worksheet “squirrel monkey samples”). DNA samples from thirty-two (32) individuals were used in this study. Various tissue and DNA samples were obtained from multiple natural science museums and research centers. Labeled biomaterials were obtained for the following squirrel monkey species: *Saimiri sciureus* (10 samples), *Saimiri sciureus sciureus* (2 samples), *Saimiri boliviensis* (14 samples), *Saimiri boliviensis peruviensis* (3 samples), *Saimiri oerstedii oerstedii* (1 sample), *Saimiri sciureus macrodon* (1 sample), and *Saimiri* “species unknown” (1 sample). DNA from tissue samples were prepared using proteinase K digestion followed by phenol: chloroform extraction and ethanol precipitation [55]. Extracted DNA was stored in 10 mM Tris/0.1 mM EDTA (TLE) and quantified spectrophotometrically using an Eppendorf Biophotometer. The DNA panel and PCR format is shown in Additional file 1.

### Polymerase chain reaction amplification

Polymerase chain reaction amplification was performed in 25 μL reactions that contained 25–50 ng of template DNA, 200 nM of each primer, 1.5 mM MgCl_2_, 10× PCR buffer, 0.2 mM deoxyribonucleotide triphosphates and 1 unit of *Taq* DNA polymerase. The polymerase chain reaction protocol is as follows: 95 °C for 1 min, 32 cycles of denaturation at 94 °C for 30 s, 30 s at the respective annealing temperature, and extension at 72 °C for 30 s, followed by a final extension step at 72 °C for 2 min. Gel electrophoresis was performed on a 2% agarose gel containing 0.2 μg/mL ethidium bromide for 60 min at 175 V. UV fluorescence was used to visualize the DNA fragments using a BioRad ChemiDoc XRS imaging system (Hercules, CA).

### *Alu* insertion polymorphisms

Following gel electrophoresis, genotypic data were recorded for each allele as follows: an individual who was homozygous present for a given *Alu* locus was assigned the code 1, 1; homozygous absent, 0, 0; and heterozygous, 1, 0. This binomial data sheet was used to calculate the allele frequency for each *Alu* insertion for the panel of 32 squirrel monkeys to evaluate the polymorphism rate. Allele frequency calculations were also performed separately for *S. sciureus* and *S. boliviensis* groups in an effort to identify species informative markers.

### DNA sequencing

PCR validation experiments identified certain ambiguous conditions that warranted further evaluation by chain termination DNA sequencing [56]. There were two basic categories; 1) gel electrophoresis revealed PCR amplicons for the predicted present / absent sizes plus a larger amplicon of unknown identity in some individuals, 2) to confirm a shared *Alu* insertion event among seemingly misidentified individuals. Sanger sequencing experiments were performed as follows: Four PCR fragments per locus were gel purified using a Wizard SV gel purification kit (Promega Corporation, Madison, WI, USA, catalog A9282) according to the manufacturer’s instructions with the following modification. The 50 μl elution step was performed twice, resulting in 100 μl, which was then dried in a SpeedVac (ThermoSavant SPD 111 V). The DNA was reconstituted in 30 μl TVLE (Tris Very Low EDTA; 10 mM Tris/ 0.05 mM EDTA) and 4 μl was used for chain termination cycle sequencing using BigDye Terminator v3.1. Cycle sequencing was performed under the following conditions: After initial denaturation at 95 °C for 2 min, 40 cycles at 95 °C for 10 s, 50 °C for 5 s, and 60 °C for 4 min were performed followed by a hold at 4 °C. Sequencing reactions were cleaned by standard ethanol precipitation to remove any unincorporated dye terminators and then stabilized in 15 μl Hi-Di Formamide (Life Technologies, Inc.). Capillary electrophoresis was performed on an ABI 3130xl Genetic Analyzer (Applied Biosystems, Inc., Foster City, CA). Sequence quality was evaluated using ABI software Sequence Scanner v.2.0. Sequencing results were then analyzed using BioEdit [52].

### Structure analysis

Population structure analyses were performed using Structure 2.3.4 software [57]. Using genotype data from unlinked markers, this software performs a model-based clustering method to infer the population structure. For our initial analysis, the information regarding the origin of the samples was omitted. The analyses were performed under the admixture model which assumes that individuals may have mixed ancestry. The settings used to determine the estimated number of populations (K) were as follows: K ranging from 1 to 7 and 10,000 burnin for 100,000 MCMC at 3 iterations. The most likely value of K was calculated to be three based on the “estimated ln probability” scores generated by Structure. Sometimes Structure detects the upper most K value. Therefore, we used Structure Harvester [58] to assess all of the likelihood values for K = 1 to 7 and determine the most likely number of population clusters. K = 2 was determined to be the best fit for the data set. Structure was then run using the following settings: K (projected number of populations) = 2; 100,000 burnin for 1 million MCMC at 5 iterations. The data from 5 iterations were averaged to generate the final data set. The final graph was generated in Excel. For comparison, a second Structure analysis was performed for K = 3 with the same parameters.

## Results

### Recently integrated *Alu* insertions

Based on a recent analysis of the genome data from Baker et al. 2017 [48], and data generated from RepeatMasker [50], we retained 48 *Alu* subfamilies in the [saiBol1] genome that contained members that were less than 2 % diverged from their respective consensus sequence. Elements that are less than 2% diverged from their consensus sequence are considered to be relatively young, as they have not accrued many mutations since their insertion [59, 60]. The data were organized in excel and sorted based on the number of elements per subfamily in various divergence categories (0.0, 0.5, 1.0, 1.5, and 2.0). These data can be found in Additional file 2. Table 1 displays the number of insertions in each divergence category. The elements descended in correlation to the divergence categories with the most elements being 2% diverged followed by 1.5, 1.0, 0.5, and 0.0% diverged. There were a total of 4184 young *Alu* elements identified in the genome having ≤2% sequence divergence from their respective consensus sequence.

**Table 1:** Number of recently integrated *Alu* elements analyzed for each percent divergence bin

Percent Divergence	Number of *Alu* Elements in [saiBol1]	Number of Loci PCR Validated	Number of Polymorphic Loci	Number of Fixed Loci
0.0	7	6	2	4
0.5	49	34	16	18
1.0	395	106	37	69
1.5	1493	168	44	124
2.0	2240	68	11	57

Total number of *Alu* insertions in the [saiBol1] genome from a range of 0% to 2% sequence divergence from their respective consensus sequence. The number of *Alu* insertions in each divergence category from the PCR validation experiments in this study is shown in the center column and separated by the number of polymorphic versus fixed loci in adjacent columns

In this study, we targeted at least *N* = 5 young insertions from each *Alu* subfamily computationally determined to contain young elements. We successfully performed PCR validation experiments on 382 *Alu* insertion events having ≤2% sequence divergence from their respective consensus sequence (Table 2) and (Additional file 1, worksheets “PCR primers & coordinates” and “genotypes”). These loci represented 46 *Alu* subfamilies, 25 from *Saimiri* specific subfamilies [48] and 21 from NWM specific subfamilies [10, 49]. On a DNA panel of 32 squirrel monkey individuals (Additional file 1, worksheet “PCR format”), 272 of the 382 loci were homozygous present for the *Alu* insertion and 110 were polymorphic for insertion presence/ absence. The number of loci analyzed per subfamily and insertion presence/absence data are listed in Table 2 and Additional file 1, worksheets “PCR primers & coordinates” and “genotypes”. The number of *Alu* insertions in each of the percent divergence bins from 0.0 to 2.0 is shown in Table 1. Table 1 illustrates that many insertions with very low sequence divergence have already reached very high allele frequency among squirrel monkey species (fixed present in our panel), while concurrently *Alu* insertions from all five divergence bins have elements that remain polymorphic in the population.

**Table 2:** PCR validation results for each *Alu* subfamily

	Subfamily	N	Fixed	Polymorphic		Subfamily	N	Fixed	Polymorphic
1	sf36	14	10	4	25	subfam15	5	4	1
2	sf37	12	10	2	26	subfam17	1	1	0
3	sf38	16	11	5	27	subfam18	1	1	0
4	sf42	24	15	9	28	subfam2	3	3	0
5	sf44	17	14	3	29	subfam21	1	1	0
6	sf46	15	9	6	30	subfam26	11	9	2
7	sf47	11	7	4	31	subfam27	1	1	0
8	sf51	16	8	8	32	subfam29	6	4	2
9	sf52	17	14	3	33	subfam30	1	1	0
10	sf53	3	2	1	34	subfam32	15	11	4*
11	sf62	12	7	5	35	subfam36	12	7	5
12	sf63	16	11	5	36	subfam37	4	4	0
13	sf65	1	1	0	37	subfam39	5	4	1
14	sf66	13	10	3	38	subfam4	7	5	2
15	sf71	14	12	2	39	subfam43	8	7	1
16	sf73	11	5	6	40	subfam45	3	3	0
17	sf82	15	10	5	41	subfam47	1	1	0
18	sf85	3	1	2	42	subfam5	9	5	4
19	sf86	11	9	2	43	subfam7	1	1	0
20	subfam0	9	6	3	44	subfam9	4	3	1
21	subfam11	3	1	2*	45	Ta10	5	5	0
22	subfam12	12	7	5	46	Ta15	5	4	1*
23	subfam13	2	2	0					
24	subfam14	6	5	1	Total		382	272	110

*Three loci in the polymorphic column, L-21071-subfam11, L-38701-subfam32 and L-19471-Ta15, were homozygous absent for the *Alu* in all 32 squirrel monkey samples on the DNA panel

The dataset of polymorphic insertions included three loci with homozygous absent genotypes (0, 0) for the target *Alu* insertion in all 32 squirrel monkey individuals: L-21071-subfam11, L-38701-subfam32 and L-19471-Ta15. These *Alu* elements were ascertained from the reference genome [saiBol1] of *S. boliviensis* but the DNA for that reference individual was not available and therefore not included on our test panel. This very low allele frequency (near zero) is indicative of very recent insertion events. These results confirm the previously reported *Alu* subfamily network analysis [48] showing the existence of many young subfamilies. These data provide evidence for a prolific expansion of young *Alu* elements in the *Saimiri* lineage currently polymorphic between species.

### Sanger sequencing validation

**Fig. 1:**
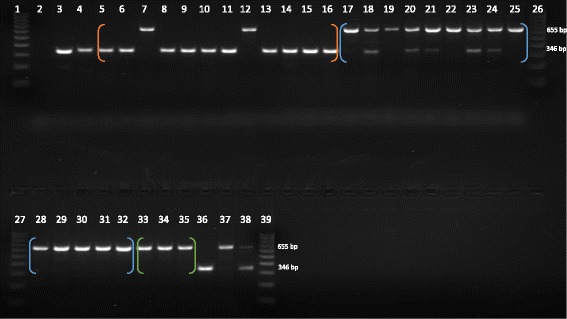
Gel Image of Polymorphic Locus 35154 (JH378108:33053451–33054957). This image displays a polymorphic locus in the *Saimiri* genome [saiBol1]. Lanes: 1- 100 bp ladder, 2- TLE (Negative control), 3- Human (HeLa), 4-Callithrix jacchus (Common marmoset), 5–16 *Saimiri sciureus* (Common squirrel monkey), 17–32 *Saimiri boliviensis* (Bolivian squirrel monkey), 33–35 *Saimiri boliviensis* peruviensis (Peruvian squirrel monkey), 36- *Saimiri oerstedii* oerstedii (Panamanian red back squirrel monkey), 37- *Saimiri sciureus* macrodon, 38-Saimiri sp. The presence of the *Alu* element is indicated by the ~ 655 bp band and the absence by the ~ 346 bp band. Species with multiple individuals are grouped together by colored brackets (Orange- Common squirrel monkey, Blue- Bolivian squirrel monkey, Green-Peruvian squirrel monkey). Lanes 7(UWBM# 75531) and 12(MVZ Mamm 193661) share an insertion with the Bolivian squirrel monkeys whom are either homozygous present or heterozygous for the insertion (lanes 17–32). Lane 38 (species unknown) is heterozygous for the insertion

During PCR validation experiments certain ambiguous conditions occurred that warranted further evaluation by traditional Sanger sequencing [56]. These conditions had two basic categories. One, gel electrophoresis revealed PCR amplicons for the predicted present / absent sizes plus a larger amplicon in some individuals. This occurred for three loci and the details are outlined in Additional file 1, Worksheet “Table S1”. DNA sequencing of the larger amplicon determined that the loci contained more than one *Alu* element or an extra *Alu* element between the original PCR primers. These non-reference (not present in the [saiBol1] genome) *Alu* insertions appeared to be polymorphic across the various species in the DNA panel. The genotypes for these three extra *Alu* polymorphisms are recorded with the locus ID –“Alu-2” in the genotypes worksheet of Additional file 1. The second category which required Sanger sequencing was to confirm a shared *Alu* insertion event among seemingly misidentified individuals. Specifically, when genotype data for individuals labeled *S. sciureus*, and believed to be common squirrel monkeys, matched more closely to the *S. boliviensis* group, sequencing was warranted. An example of this is shown in Fig. 1. Forty-five loci from the dataset of 382 matched this condition. We sequenced 28 of the 45 and it was determined that all amplicons except one were the same *Alu* element identified in the reference genome. Only one amplicon was a near parallel insertion (Locus 16089, individual UWBM# 75531).

### Population structure

Following PCR and gel electrophoresis, genotypes for the 32 squirrel monkey individuals were recorded in an excel spreadsheet as follows: homozygous absent for the reference *Alu* insertion, (0, 0), homozygous present for the target *Alu* insertion (1, 1) and heterozygous as (1, 0) (Additional file 1, worksheet “genotypes”). During genotype analysis we identified 24 loci (of 382) with > 25% missing data due to poor PCR (highlighted in tan in the genotype spreadsheet; Additional file 1). Most of these (21) were homozygous present for the insertion and would not influence population structure, but 3 were from the polymorphic dataset. These were omitted from the population structure analysis. Also, the samples from the KCCMR *S. boliviensis* breeding colony included two known sibling pairs, one sibling from each sibling pair was omitted from the Structure analysis [57].

**Fig. 2:**
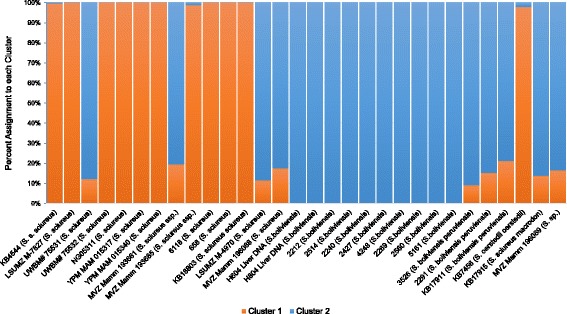
Population Structure analysis based on 110 *Alu* insertion polymorphisms and 32 squirrel monkey individuals for K = 2. The percent assignment of each individual to K = 2 clusters is shown on the Y-axis. The ID numbers and species names are shown on the X-axis. K = 2 captures the population structure of the two *Saimiri* groups, *S. sciureus* and *S. boliviensis*, and is consistent with the geographic origins of these samples

To determine the value of K (where K equals the number of population clusters) with the highest likelihood, initially K was set from 1 to 7. The initial burn-in period was set at 10,000 iterations and followed by a run-length of 100,000 MCMC and repeated three times. The most likely value of K was calculated to be three based on the “estimated ln prob. of data” scores generated by Structure. The authors of Structure indicate that this method is generally accurate with small data sets, but acknowledge it is still an estimate of K. Therefore, we also employed the Delta K method by Evanno [61] implemented using Structure Harvester [58]. The Delta K method is widely accepted in the literature as an accurate estimate of the true K. Here, the Delta K was calculated to be K = 2. The structure results for K = 2 are shown in Fig. 2. In general, Cluster 1 contains individuals previously labeled as common squirrel monkeys and Cluster 2 contains individuals previously labeled as Bolivian squirrel monkeys. However, there is a large amount of admixture in some individuals (a mixture of Cluster 1 & 2). These admixed appearing individuals were previously labeled as common squirrel monkey (UWBM # 75531 & MVZ Mamm 193661), Bolivian squirrel monkey (LSUMZ M-4970, MVZ Mamm 196088), Peruvian squirrel monkey (3526, 2291, KB17911), Ecuadorian squirrel monkey (KB17915) and species unknown (MVZ Mamm 196089). The results of this Structure analysis are generally consistent with the geographic ranges of the *Saimiri* species and subspecies. Maps of the geographic ranges can be found in Hershkovitz 1984 and Chiou et al. 2011 [39, 40]. Sample KB7456 is the only member of the *S. oerstedii* species on our panel. This species is the Panamanian squirrel monkey located in Central America. The geographic range of *S. oerstedii* is closer to the *S. sciureus* group than to the *S. boliviensis* group [39, 40] and Structure assigns this individual to Cluster 1. The geographic “locality” provided for sample MVZ Mamm 193661 (clusters with Bolivian) is the Acre region of Brazil (listed in Additional file 1, worksheet “Squirrel monkey samples”) and it is labeled *Saimiri sciureus ssp. the* Acre region is consistent with the geographic range of *S. sciureus macrodon* and borders the region of *S. boliviensis peruviensis* [39]. *S. sciureus macrodon* are the Ecuadorian squirrel monkeys native to Peru. Therefore, we can interpret these results as meaning that MVZ Mamm 193661 has an incomplete identification, rather than being misclassified. MVZ Mamm 193685 was also labeled as *Saimiri sciureus ssp. the* geographic locality provided for this sample is the Penedo region of Brazil, consistent with the geographic range of *Saimiri sciureus sciureus,* and consistent with the Structure assignment to Cluster 1. MVZ Mamm 196089 is labeled *Saimiri sp.,* indicating the species is not known. The geographic locality listed for this sample is the Sao Jose region of Brazil, the same locality as reported for MVZ Mamm 196088, and consistent with the geographic range of *S. boliviensis.* Therefore, we can infer that this dataset accurately captures the majority of the geographic population structure among *Saimiri* species.

**Fig. 3:**
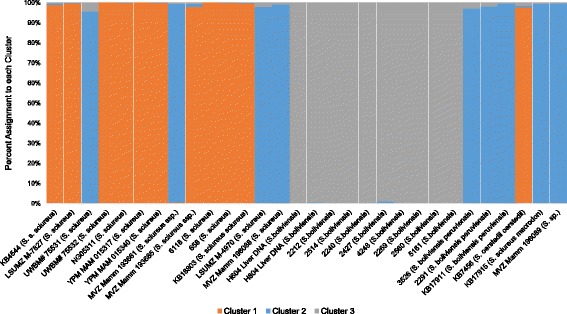
Population Structure analysis based on 110 Alu insertion polymorphisms and 32 squirrel monkey individuals for K = 3. The percent assignment of each individual to K = 3 clusters is shown on the Y-axis. The ID numbers and species names are shown on the X-axis. K = 3 captures the population structure of the two Saimiri groups, *S. sciureus* and *S. boliviensis* while also detecting the genetic isolation of members of a captive breeding colony within the *S. boliviensis* samples

However, the original “estimated ln prob. of data” scores generated by Structure suggested that K = 3 was likely. In an effort to make sure our interpretations of the data were accurate, we also tested K = 3 (Fig. 3 and Additional file 2, sheet K = 3 Table). In Fig. 3, the samples from JAV (DNA originally from KCCMR) and KCCMR appear to be isolated and more genetically similar. If the dataset is analyzed using K = 3, the third cluster is formed by isolating the ten members in the dataset from the KCCMR *S. boliviensis* captive breeding colony into its own cluster (shown in gray in Fig. 3), the remaining individuals segregate into the other two clusters similar to their respective assignments in the K = 2 analysis (orange and blue). To further investigate this observation we analyzed the Fst values for K = 2 and 3 for all of the clusters (Table 3). When K = 2 Fst values are similar, which implies the populations share genetic diversity. When K = 3 two clusters have similar values and one cluster has an extremely low value of 0.3391. A value of 0.3391 would imply that the individuals in Cluster 3 may be sharing genetic material through high levels of inbreeding and appears to be an isolated group in Fig. 3. While K = 2 captures the primary geographic origins of the *Saimiri* populations, K = 3 is also reasonable as it reveals genetic evidence of inbreeding among members of a captive colony.

**Table 3:** Average Fst Values for K = 2 and K = 3

K Value	Cluster Number	Average Fst
K = 2	1	.7747
K = 2	2	.6950
K = 3	1	.8014
K = 3	2	.7639
K = 3	3	.3391

Average Fst values for K (estimated population clusters) equals 2 and K equals 3. If K = 2, Fst values are similar which implies genetic similarity between populations. If K = 3, Fst values are similar for two population clusters and one cluster has an extremely low value of 0.3391. That extremely low value implies Cluster 3 is sharing genetic material through inbreeding and appears to be isolated

### Species informative *Alu* polymorphisms

Within the dataset of 110 polymorphic *Alu* insertions, there were 51 with species informative allele frequency distribution between *Saimiri sciureus* and *Saimiri boliviensis*. A locus was categorized as species informative if it was present at a high frequency in one species and generally absent in the other. These are listed in Table 4 and are highlighted in green in Additional file 1, Worksheets “PCR primers & coordinates” and “Genotypes”. The 14 members of *S. boliviensis* have a group allele frequency of 80–100% whereas the 12 samples labeled *S. sciureus* have a group allele frequency of 0–20% on average (Table 4). If we omit samples 75531 and 193661 from the *S. sciureus* group due to the Structure data (described above) showing that these two samples justifiably clustered more closely with the *S. boliviensis* group, then the group allele frequency in the *S. sciureus* group drops to near zero (0.5% on average) (Table 4). These 51 *Alu* insertion events represent 26 different *Alu* subfamilies, 10 *Saimiri* lineage specific subfamilies reported in Baker et al. 2017 [48] and 12 NWM *Alu* subfamilies discovered previously in Marmoset [49].

**Table 4:** Allele frequency data for *Alu* insertions with species informative distributions

		a. *N* = 12	b. *N* = 14	c. *N* = 10
	*Alu* Locus Name	*Saimiri sciureus*	*Saimiri boliviensis*	*Saimiri sciureus*
1	L-20858-sf38	0.000	0.893	0.000
2	L-40335-subfam32	0.000	0.893	0.000
3	L-21370-subfam26	0.083	1.000	0.000
4	L-26673-subfam29	0.167	0.857	0.000
5	L-16089-Subfam26	0.167	1.000	0.050
6	L-27488-subfam4	0.167	1.000	0.000
7	L-27102-subfam5	0.083	0.929	0.000
8	L-29927-Subfam4	0.150	0.964	0.056
9	L-22568-sf37	0.167	0.929	0.000
10	L-18103-subfam11	0.125	0.964	0.050
11	L-11426-sf51	0.182	1.000	0.000
12	L-14471-sf63	0.083	0.964	0.000
13	L-19033-sf66	0.000	0.833	0.000
14	L-12684-sf63	0.000	0.786	0.000
15	L-1748-subfam0	0.167	1.000	0.000
16	L-13945-sf46	0.042	1.000	0.000
17	L-20802-sf62	0.167	1.000	0.000
18	L-17843-sf62	0.167	0.913	0.000
19	L-6918-subfam43	0.208	0.964	0.050
20	L-31469-subfam29	0.042	0.929	0.000
21	L-24998-subfam36	0.000	1.000	0.000
22	L-40504-sf42	0.167	1.000	0.000
23	L-26020-sf85	0.167	1.000	0.000
24	L-33213-sf86	0.167	1.000	0.000
25	L-2485-sf82	0.042	1.000	0.000
26	L-35028-sf63	0.125	1.000	0.000
27	L-18718-sf62	0.167	1.000	0.000
28	L-6892-sf71	0.167	1.000	0.000
29	L-7578-sf82	0.167	1.000	0.000
30	L-19942-sf73	0.167	1.000	0.000
31	L-20830-sf73	0.200	1.000	0.000
32	L-25034-subfam36	0.167	0.923	0.000
33	L-38119-subfam12	0.167	0.964	0.000
34	L-30099-sf52	0.167	1.000	0.000
35	L-36916-subfam12	0.208	1.000	0.050
36	L-8051-sf42	0.167	1.000	0.000
37	L-24655-s42	0.167	0.964	0.000
38	L-39021-sf51	0.167	1.000	0.000
39	L-16832-sf82	0.083	0.929	0.000
40	L-20778-sf73	0.167	1.000	0.000
41	L-37765-sf82	0.111	1.000	0.000
42	L-30633-sf86	0.125	1.000	0.000
43	L-431-sf66	0.125	1.000	0.000
44	L-20383-sf36	0.167	1.000	0.000
45	L-30828-subfam5	0.167	0.893	0.000
46	L-22291-sf46	0.125	0.893	0.000
47	L-25257-sf42	0.167	1.000	0.000
48	L-26813-sf42	0.125	1.000	0.000
49	L-28766-sf38	0.167	1.000	0.000
50	L-38773-sf44	0.167	1.000	0.000
51	L-10445-sf46	0.000	0.857	0.000

Allele frequency data for 51 polymorphic *Alu* insertions with species informative distribution between *S. sciureus* and *S. boliviensis* squirrel monkey species. Column C. with only ten *S. sciureus* samples has #75531 and #193661 omitted from the calculation because they clustered more closely with the Bolivian cluster (See Fig. 2). The 14 *S. boliviensis* group have an allele frequency of 80–100% whereas the 12 samples labeled *S. sciureus* have a group allele frequency of 0–20%. With #75531 and # 193661 omitted in column C, the group allele frequency in the *S. sciureus* group drops to near zero (0.5% on average). These 51 *Alu* insertion polymorphisms represent 26 different subfamilies: 10 *Saimiri* lineage specific *Alu* subfamilies reported in Baker et al. 2017 [48] and 16 NWM *Alu* subfamilies discovered in marmoset [49]

## Discussion

An analysis of a large number of *Alu* insertions from many different *Alu* subfamilies, and a diverse DNA panel of squirrel monkeys, allowed us to determine the *Alu* insertion diversity in the *Saimiri* lineage. This suggests that many different *Alu* subfamilies were active in *Saimiri* and generated new *Alu* insertions. These data also support the stealth model of *Alu* amplification [62] in which relatively older *Alu* subfamilies are still producing new copies. In this case, the *Alu*Ta subfamily [63] is estimated to have originated about 15 MYA).

However, this study also has limitations, considering only one *Saimiri* species has a sequenced genome, *S. boliviensis*. The *Alu* elements in this study were ascertained from the reference genome [saiBol1] of a Bolivian squirrel monkey. The allele frequency data for the polymorphic insertions reflect the inherent single genome frequency spectrum ascertainment bias. Within the dataset of 51 polymorphic *Alu* insertions with species informative allele frequency distribution between *Saimiri sciureus* and *Saimiri boliviensis* samples, the *S. boliviensis* group has a relatively high allele frequency (~ 80–100%) whereas the *S. sciureus* group has a very low allele frequency (near zero) (Table 4). However, the three new polymorphic *Alu* insertions discovered during Sanger sequencing appear to be *S. sciureus* derived, rather than *S. boliviensis* derived (Additional file 1, genotypes worksheet). As more whole genome sequence data become available for *Saimiri* species, the frequency spectrum limitation due to ascertainment from a single reference genome will diminish. Thus, a more comprehensive assessment of *Alu* mobilization dynamics among *Saimiri* species will be attainable.

Prior to 1984, squirrel monkeys were considered a single species, named *Saimiri sciureus*, with many subspecies geographically separated [28]. Therefore, it is not surprising that some archival tissue samples from natural science museums or specimens from older studies may have typically been labeled simply as *Saimiri*, squirrel monkey, or *S. sciureus*. This does not mean they are necessarily mislabeled, but more than likely represent incomplete identification due to limited availability regarding source animal data at the time of sampling. Although we have no direct confirmation that this occurred with some of the samples in our DNA panel, the genetic diversity evidence from the Structure analysis in this study suggests it is likely. Individuals UWBM#75531, MVZ Mamm 193661 and MVZ 196089 in particular had ambiguous amplicons in 45 different *Alu* loci. Based on our Sanger sequencing, geographic locality and the Structure data, we believe these individuals may have previously been “under-classified” and they are most closely related to the Ecuadorian squirrel monkeys, *S. sciureus macrodon,* or the Peruvian squirrel monkeys *S. boliviensis peruviensis*. Considering there were only three Peruvian squirrel monkeys and one Ecuadorian squirrel monkey on the DNA panel, a larger sample size with more whole genome sequence data would be required for the identification of the exact species of these individuals.

## Conclusions

Many different *Alu* subfamilies were active in the *Saimiri* genome producing a large number of young polymorphic insertions. These young polymorphic *Alu* insertions provide a valuable resource for species identification and population structure within *Saimiri*. This dataset may prove useful to natural science museums that may contain archival tissue samples labeled simply as “*Saimiri*” or “squirrel monkey” due to limited data available about the source animal at the time of sampling. Some of these samples may now be further classified at the species level and possibly even at the subspecies level, with this dataset. Future whole genome sequencing studies will further elucidate these findings.

## Additional files

Additional file 1: An excel file containing worksheets for PCR primers & coordinates, Squirrel monkey samples, PCR format, and genotype data for each locus. Table S1 details the loci sequenced. (XLSX 503 kb)

Additional file 2: An excel file showing *Alu* element subfamilies in the [saiBol1] genome with 0% to 2% divergence from their respective consensus sequences and the number of members per divergence category. A separate worksheet shows the numerical values for K = 3 Structure analysis. (XLSX 19 kb)

## Abbreviations

Bp: Base pair
NWM: New World Monkey
PCR: Polymerase chain reaction
SINE: Short interspersed element
UCSC: University of California Santa Cruz
